# Computerized Decision Support for Bladder Cancer Treatment Response Assessment in CT Urography: Effect on Diagnostic Accuracy in Multi-Institution Multi-Specialty Study

**DOI:** 10.3390/tomography8020054

**Published:** 2022-03-02

**Authors:** Di Sun, Lubomir Hadjiiski, Ajjai Alva, Yousef Zakharia, Monika Joshi, Heang-Ping Chan, Rohan Garje, Lauren Pomerantz, Dean Elhag, Richard H. Cohan, Elaine M. Caoili, Wesley T. Kerr, Kenny H. Cha, Galina Kirova-Nedyalkova, Matthew S. Davenport, Prasad R. Shankar, Isaac R. Francis, Kimberly Shampain, Nathaniel Meyer, Daniel Barkmeier, Sean Woolen, Phillip L. Palmbos, Alon Z. Weizer, Ravi K. Samala, Chuan Zhou, Martha Matuszak

**Affiliations:** 1 Department of Radiology, University of Michigan, Ann Arbor, MI 48109, USA; lhadjisk@med.umich.edu (L.H.); chanhp@med.umich.edu (H.-P.C.); rcohan@med.umich.edu (R.H.C.); caoili@med.umich.edu (E.M.C.); matdaven@med.umich.edu (M.S.D.); pshankar@med.umich.edu (P.R.S.); ifrancis@med.umich.edu (I.R.F.); kshampai@med.umich.edu (K.S.); nbmeyer@med.umich.edu (N.M.); dbarkmei@med.umich.edu (D.B.); sean.woolen@ucsf.edu (S.W.); rsamala@med.umich.edu (R.K.S.); chuan@med.umich.edu (C.Z.); 2 Department of Internal Medicine-Hematology/Oncology, University of Michigan, Ann Arbor, MI 48109, USA; ajjai@med.umich.edu (A.A.); ppalmbos@med.umich.edu (P.L.P.); 3 Department of Internal Medicine-Hematology/Oncology, University of Iowa, Iowa, IA 52242, USA; yousef-zakharia@uiowa.edu (Y.Z.); rohan-garje@uiowa.edu (R.G.); dean-elhag@uiowa.edu (D.E.); 4 Department of Internal Medicine-Hematology/Oncology, Pennsylvania State University, Hershey, PA 16801, USA; mjoshi@pennstatehealth.psu.edu (M.J.); lpomerantz@pennstatehealth.psu.edu (L.P.); 5 Department of Neurology, University of Michigan, Ann Arbor, MI 48109, USA; kerrwe@med.umich.edu; 6 U.S. Food and Drug Administration, Center for Devices and Radiological Health, Silver Spring, MD 20993, USA; kenny.cha@fda.hhs.gov; 7 Department of Radiology, Acibadem City Clinic, Tokuda Hospital, 1407 Sofia, Bulgaria; gal.kirova@gmail.com; 8 Department of Urology, University of Michigan, Ann Arbor, MI 48109, USA; aweizer@med.umich.edu; 9 Department of Radiation Oncology, University of Michigan, Ann Arbor, MI 48109, USA; marthamm@med.umich.edu

**Keywords:** observer study, computer-aided diagnosis, bladder cancer, treatment response

## Abstract

This observer study investigates the effect of computerized artificial intelligence (AI)-based decision support system (CDSS-T) on physicians’ diagnostic accuracy in assessing bladder cancer treatment response. The performance of 17 observers was evaluated when assessing bladder cancer treatment response without and with CDSS-T using pre- and post-chemotherapy CTU scans in 123 patients having 157 pre- and post-treatment cancer pairs. The impact of cancer case difficulty, observers’ clinical experience, institution affiliation, specialty, and the assessment times on the observers’ diagnostic performance with and without using CDSS-T were analyzed. It was found that the average performance of the 17 observers was significantly improved (*p* = 0.002) when aided by the CDSS-T. The cancer case difficulty, institution affiliation, specialty, and the assessment times influenced the observers’ performance without CDSS-T. The AI-based decision support system has the potential to improve the diagnostic accuracy in assessing bladder cancer treatment response and result in more consistent performance among all physicians.

## 1. Introduction

In 2021, it was estimated that about 83,730 new cases of bladder cancer would be diagnosed and about 17,200 would die from it in the US. This would account for 0.4% of all new cancer cases and 2.8% of all cancer deaths [1]. The 5-year relative survival rate was 96% for in situ stage, 69% for localized stage, 37% for regional stage, and 6% for distant stage. The 5-year relative survival rate for all SEER stages combined is 77% [1]. Early diagnosis can improve the survival rate. Neoadjuvant chemotherapy performed prior to radical cystectomy can improve patient survival rate and decrease the probability of metastatic disease [2,3,4]. However, neoadjuvant chemotherapy has toxicities including neutropenic fever, sepsis, mucositis, nausea, vomiting, malaise, and alopecia [5]. It is of great importance to evaluate the response of bladder lesions to chemotherapy treatment to spare the patient the toxicities of further unnecessary chemotherapy or to support surgery de-escalation [6].

We have developed a computerized artificial intelligence (AI)-based decision support system for muscle-invasive bladder cancer treatment response assessment (CDSS-T) to assist physicians to evaluate the response to treatment of these cancers on pre- and post-treatment CT urography (CTU) scans [7]. It is critical to gain understanding of various factors that may affect the impact of CDSS-T on physician performance in identifying bladder cancers with complete response after neoadjuvant chemotherapy through observer studies that can guide the design of future clinical trials. Patients with complete response may be considered for organ preservation therapy instead of cystectomy (the removal of the bladder). Our previous studies showed that CDSS-T can improve physician performance from a single institution [6,8,9]. The goal of the current study is to further investigate the impact of CDSS-T on the performance of physicians from different specialties and different institutions.

## 2. Materials and Methods

### 2.1. Data Set

With Institutional Review Board (IRB) approval, we collected the pre- and post-chemotherapy CTU scans of 123 patients (157 pre- and post-treatment cancer pairs). The pathological cancer stage after treatment was collected as reference standard for determining if a patient had complete response to treatment. The reference standard indicated that 40 out of 157 lesion pairs had complete response (stage T0) after chemotherapy, and 117 lesion pairs had incomplete response (>T0) after chemotherapy. The patient and cancer characteristics are presented in Table 1.

### 2.2. Computerized AI-Based Decision Support System for Treatment Response Assessment (CDSS-T)

CDSS-T combined a deep-learning convolutional neural networks (DL-CNN) model and a radiomics model to estimate the likelihood of response to neoadjuvant chemotherapy treatment [7]. Each model output a discriminant score for each scan pair. A combined score was generated by taking the maximum of the two scores [7].

### 2.3. DL-CNN Assessment Model

Bladder cancers on the CTU images were segmented using the auto-initialized cascaded level sets (AL-CALS) system [10]. Multiple regions of interest (ROIs) were extracted from the segmented tumor slices. The size of the ROIs was 32 × 16 pixels. A hybrid ROI of 32 × 32 pixels was composed using an ROI from the pre-treatment scan and an ROI from the post-treatment scan of the same tumor. Multiple hybrid ROIs were generated from the same pre- and post-treatment CTU scan pairs by taking different combinations of the pre- and post-treatment ROIs [7]. All hybrid ROIs were labeled as a complete responder (T0) or a non-complete responder (>T0) based on the pathological stage of the cancer after treatment. The hybrid ROIs were used to train and test the DL-CNN model using a leave-one-case-out cross-validation scheme. The DL-CNN model was trained with all hybrid ROIs except for those from the left-out case. The trained DL-CNN model was then applied to the hybrid ROIs of each left-out test case, and a likelihood score of pathologic T0 disease was output for each test ROI. The average value of all the hybrid ROIs associated with the CT scan pair of a specific cancer was the discriminant score for this cancer.

### 2.4. Radiomics Assessment Model

A radiomics-feature-based analysis was used for this assessment model. Ninety-one features were extracted from each CTU scan [7]. The chosen features had previously been demonstrated to be useful in analysis of breast mass [11], lung nodules [12], and bladder cancer treatment response assessment [7]. For each cancer, the features from the pre- and post-treatment scan were compared and the percent difference of each radiomics feature was calculated. To build this model, a two-loop leave-one-case-out cross-validation scheme [13] was used. For the inner loop, the subset of features was selected, and the random forest classifier [7] was trained by using a leave-one-case-out scheme within the training partition. For the outer loop, the trained classifier was applied to the left-out test case and a discriminant score was generated by the radiomics model. An average of four features was selected, including two run-length statistics features and two contrast features.

### 2.5. CAD Score

**Figure 1:**
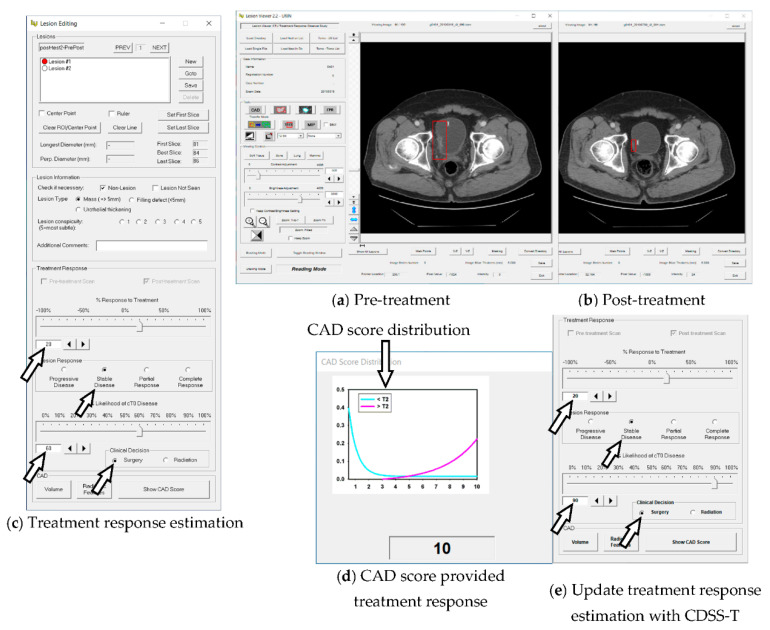
The graphical user interface for reading with and without our computerized decision support system (CDSS-T) for bladder cancer treatment response assessment. (**a**,**b**) The pre- and post-treatment CTU scans are shown side-by-side. (**c**) The observer estimates the treatment response. (**d**) The observer is shown the CAD score and the score distribution of the two classes as reference. (**e**) The observer may revise their treatment response assessment after considering the CAD score.

For each pre-post-treatment scan pair, a combined score was obtained by taking the larger value of the two discriminant scores generated by the DL-CNN model and the radiomics model. Receiver operating characteristics (ROC) analysis was performed on the combined scores to estimate the CDSS-T performance. Linearly scaling the combined scores generated computer-aided diagnosis (CAD) scores in the range from 1 to 10. Smaller scores indicated lower probability that the post-treatment lesion had a complete response, and larger scores indicated higher probability that the post-treatment lesion had a complete response A curve was fitted to the distribution of the linearly transformed scores for the cancers in each class, i.e., the stage T0 and the stage > T0 cancers. The area under each fitted curve was normalized to 1. The CAD score for the cancer being read and the two fitted curves were displayed to the observers during the CDSS-T assisted reading. The fitted distributions provided a reference of the likelihood of complete response for the CAD score (Figure 1d). The CAD scores and the fitted curves were predetermined and were the same for all observers.

### 2.6. Observer Performance Study

Seventeen physicians from six specialties and four institutions, University of Michigan (UM), Pennsylvania State University (PSU), University of Iowa (UI), and Tokuda Hospital (TH), Sofia, provided assessment of response by reading the pair of pre- and post-treatment CTU for each cancer. The specialty and institution information of observers is presented in Table 2. A graphical user interface developed for CDSS-T was used to assist physicians to make the estimations. **First, without CDSS-T** (Figure 1a,b), the pre- and post-treatment scans were shown side-by-side. The physician provided the estimation of treatment response (Figure 1c), consisting of: (1) percentage response to treatment on a scale of −100% to +100% using the Response Evaluation Criteria in Solid Tumors (RECIST) criteria [14], where 0% indicated no change between the pre- and post-treatment scan, −100% indicated at least doubling of tumor size, and 100% indicated a complete response; (2) tumor response category (based on the RECIST criteria) including progressive disease, stable disease, partial response, and complete response [14]; (3) likelihood of T0 stage (complete response) on a scale of 0 to 100%, where 0 indicated no chance of this post-treatment cancer being at T0 stage and 100% indicated 100% chance of this post-treatment cancer being at T0 stage; (4) a recommendation for the next treatment procedure including surgery or radiation. **Second, with CDSS-T** (Figure 1d), a CAD score along with the CAD score distributions was shown to the physician. The physician might revise his/her estimates or leave them unchanged (Figure 1e). For the analysis of intra-observer variability, a subset (mean *N* = 51) of the 157 lesion pairs was assessed for the second time by the 17 observers. Note that this subset or 51 lesions was randomly sampled so that it varied for each observer. The first read of the subset was called the original evaluation, and the second read of the subset was the repeated evaluation in the following discussion.

### 2.7. Statistical Analysis

The physicians’ estimates of the likelihood of T0 stage (complete response) were analyzed with multi-reader, multi-case (MRMC) ROC methodology [15]. The MRMC analysis tool can provide values of area under curve (AUC) and numerical information which can be used to plot ROC curves. A *p*-value of less than 0.05 was considered to indicate a significant difference.

A number of comparisons were performed, including between observers with different degrees of clinical experience, observers from different institutions, observers from different specialties, and between two subsets of cases with different levels of diagnostic difficulty. The intra-observer variability was estimated from the repeated readings of 51 cases. As for the degree of experience, the radiologists, oncologists, and urologists were regarded as experienced physicians in assessing bladder cancer treatment response, and the radiology residents and the neurology fellow were regarded as inexperienced physicians. Note that the medical student was also categorized as an “inexperienced physician” for simplicity of discussion. For each cancer, the standard deviation of the estimates of likelihood of T0 stage by nine radiologists was used to evaluate the level of difficulty in assessing the response of the cancer. The assessment was categorized as relatively easy when the standard deviation was less than 25, and the corresponding scan pair was grouped into the “easy cancer” subset, or relatively difficult when the standard deviation was larger than 25, and the corresponding scan pair was grouped into the “difficult cancer” subset. The threshold of 25 was selected by approximately balancing the number of complete responses (T0 after chemotherapy) in the easy and difficult cancer subsets for the purpose of performing a reliable ROC analysis [6]. There were 95 pre-post-treatment scan pairs in the easy subset, in which 77 cancers did not respond completely (>T0) and 18 had a complete response (T0). The remaining 62 pairs were grouped in the difficult subset, with 40 cancers not responding completely and 22 with complete response.

The physicians’ estimates of the likelihood of T0 stage of the original and repeated evaluations were not fully-crossed due to the varied subset for different observers. We used the iMRMC package [16] for the analysis of the intra-observer variability which can handle the data and output the AUCs and the statistical significance of the difference in the AUCs [16]. The Bland–Altman method and the Krippendorff’s alpha method were also employed to evaluate the intra-observer variability.

The Krippendorff’s alpha method assesses the agreement between two response outcomes from multiple cases and multiple observers, i.e., inter-observer variability, and allows for missing responses for the cases. We applied the Krippendorff’s alpha method to the estimates of the 17 observers on the 157 cases. Krippendorff suggests 0.8 as a customary threshold for satisfactory reliability, but if tentative conclusions are acceptable, 0.667 is the lowest conceivable limit [17].

## 3. Results

### 3.1. Overall Results for All Cancers

**Figure 2:**
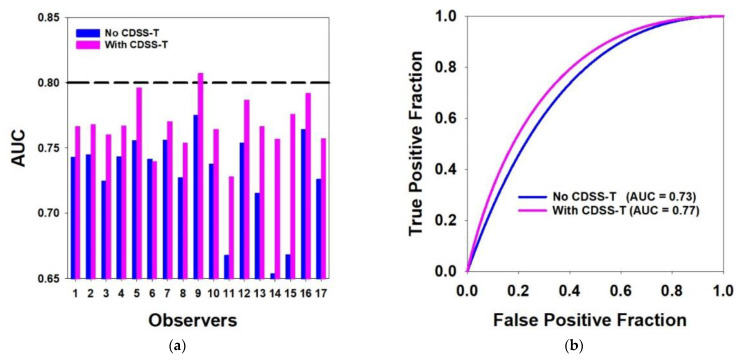
Results of the observer performance study. (**a**) AUCs of the 17 observers with and without CDSS-T. The performance of the CDSS-T system is shown with the dashed line. The performance of all but one observer (#6) increased with using CDSS-T. (**b**) Average ROC curves with and without CDSS-T.

The AUC of the CDSS-T CAD scores was 0.80. The average ROC curves with and without CDSS-T aid for all observers together with the individual AUC values with and without CDSS-T aid for the 17 observers are presented in Figure 2. The individual *p* values are presented in Table 3. All observers except #6 had larger AUCs with the CDSS-T aid (Figure 2), but the improvement reached statistical significance only for 7 of the 17 observers (*p* values in Table 3). Observer #9 with CDSS-T aid had a larger AUC (0.81) than CDSS-T alone (0.80), but the difference did not achieve significance (*p* = 0.776). The average AUC over all observers was 0.73 without CDSS-T and improved to 0.77 with CDSS-T. The difference was statistically significant (*p* = 0.002).

### 3.2. Easy vs. Difficult Cancer Subsets

The AUC of the CDSS-T was 0.88 for the easy subset, and 0.67 for the difficult subset. The performance comparisons of observers for the easy subset and the difficult subset are shown in Table 4. All 17 physicians, and the 9 radiologists, achieved statistically significant improvement for the easy subset (*p* < 0.035), but had no significant improvement for the difficult subset (*p >* 0.148). The five oncologists had significant improvement for the difficult subset (*p* = 0.009), but a borderline significance for the easy subset (*p* = 0.051).

### 3.3. Experienced vs. Inexperienced Observers

The performance comparisons of experienced and inexperienced physicians are shown in Table 5. We can see there was no observable difference between their performances. The level of statistical significance of the inexperienced radiologists was slightly higher (*p* = 0.007) after using CDSS-T compared to that of experienced radiologists (*p* = 0.06). The use of CDSS-T resulted in more consistent performance among all subgroups of physicians (all AUC = 0.77).

### 3.4. Multi-Specialty Observers

**Figure 3:**
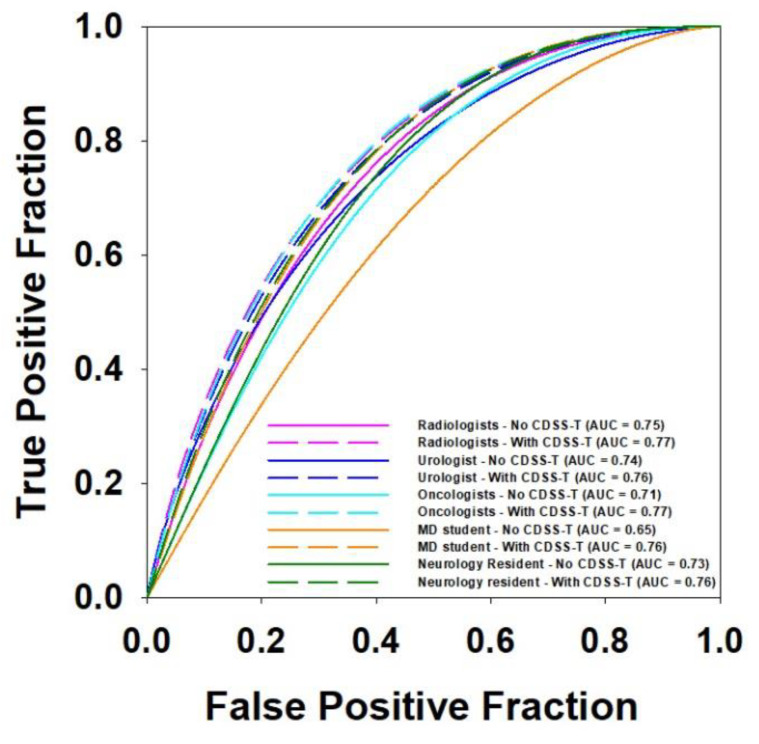
ROC curves comparison for observers of different specialties.

The average AUC values and the corresponding ROC curves for the physicians from the different specialties are shown in Table 6 and Figure 3, respectively. Radiologists, oncologists, and the medical student had statistically significant improvement after using CDSS-T (*p* < 0.020). The improvement gained by the urologist and the neurology fellow did not reach statistical significance (*p* = 0.244 and *p* = 0.083, respectively). Note that the number of observers in each group was different so that the level of significance should not be directly compared. There was a difference between the average AUCs of observers from different specialties without CDSS-T. However, with CDSS-T the performance was improved to a similar level.

### 3.5. Multi-Institution Observers

**Figure 4:**
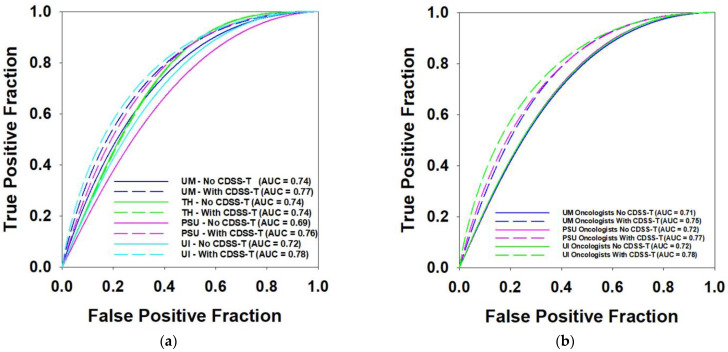
ROC curves comparison for observers from different institutions. (**a**) Performance of observers from four institutions. (**b**) Performance of oncologists from three institutions.

The average AUC values and the corresponding ROC curves of observers from different institutions are shown in Table 7 and Figure 4, respectively. The UM physicians (all specialties) and the PSU oncologist achieved statistically significant improvement with CDSS-T. Again, note the varied number of observers in the different groups so that the level of significance should not be compared directly.

### 3.6. Inter- and Intra-Observer Variability

The individual AUC values and the standard deviations of 17 observers for the original and the repeated evaluation are presented in Table 8. For the original evaluation, the average AUC increased significantly from 0.75 (without CDSS-T aid) to 0.81 (with CDSS-T aid) (*p* = 0.003). For the repeated evaluation, the average AUC also increased significantly from 0.77 (without CDSS-T aid) to 0.81 (with CDSS-T aid), (*p* = 0.006). There was no significant difference between the average AUC values for the original and repeated evaluations without CDSS-T (*p* = 0.217) or for the evaluations with CDSS-T (*p* = 0.692). The standard deviation of the observers’ results was slightly smaller with CDSS-T.

#### 3.6.1. Bland–Altman Analysis

The standard deviation of the original and repeated evaluations of each observer was calculated by the Bland–Altman method, without and with CDSS-T aid, respectively. The mean value of the standard deviations for the 17 observers was 25.47 without CDSS-T and was reduced to 19.72 with CDSS-T.

#### 3.6.2. Krippendorff’s Alpha Method

The agreement between the original and repeated evaluations of each of the observers without and with CDSS-T aid was calculated by the Krippendorff’s alpha method. The average of the 17 Krippendorff’s alpha reliability coefficients was 0.69 without CDSS-T and increased to 0.81 with CDSS-T.

The Krippendorff’s alpha reliability coefficient of the 17 observers for the whole data set (*N* = 157) was 0.56 without CDSS-T and increased to 0.67 with CDSS-T.

## 4. Discussion

For the overall performance evaluation (17 observers and 157 cancers), the results showed that the CDSS-T aid can improve the observers’ performance significantly in assessing bladder treatment response. The diagnostic difficulty of cancer cases can have an impact on the performance as seen in Table 4. However, the increases in AUC between the easy and difficult subsets were essentially equal for most of the groups in Table 4, except for the group of UM radiologists that showed a 0.01 larger improvement for the easy subset than the difficult subset. While the other groups had significant improvement for easy subset but not for difficult subset, the five oncologists only had significant improvement for difficult subset, from AUC of 0.57 without CDSS-T to 0.63 with CDSS-T. This happened because only oncologists reached AUC of 0.63 with CDSS-T which was the best, while the other two groups were 0.60~0.62, and it increased from AUC of 0.57 which was the lowest AUC score without CDSS-T among the four groups. The level of physician experience only contributed to a small difference in the level of improvement (a difference of 0.01) in assessing cancer treatment response. Without CDSS-T, it may be expected that the performance of experienced observers would be better than that of inexperienced observers, as can be observed from the slightly higher AUCs of 0.74 and 0.75 in Table 5. With CDSS-T, the experienced and inexperienced observers reached the same average AUC level (0.77). The smaller gain in AUC by the experienced observers may reflect that they were more confident and persistent in their own assessments. More importantly, the CDSS-T aid was able to assist the inexperienced observers in making diagnosis at a level comparable to that of experienced observers.

The performance of observers from the different specialties without CDSS-T did differ. In this study, on average, oncologists had a larger gain in AUC than radiologists with the CDSS-T aid, suggesting that CDSS-T could be a potentially useful tool for non-radiology physicians. There was some difference in the improvement of observers from different institutions.

For both the original and repeated evaluations, the CDSS-T aid can improve the observers’ performance significantly. The CDSS-T system can also narrow the performance gap between observers, as demonstrated by the Krippendorff’s alpha reliability coefficients of the 17 observers without and with CDSS-T. With CDSS-T aid, the observers had less variability and better agreement for both the original and repeated evaluations.

There were limitations in our study. In some of the experiments, the lack of statistical significance may be due to the small number of observers. For example, we had only one urologist and one neurology resident, and their individual performance was not significantly improved with the CDSS-T aid although they achieved substantial improvement. Additionally, the number of cases was relatively small. Leave-one-case-out cross-validation method was used to generate the CAD score. The AUC of the CDSS-T system was 0.80 which was higher than the majority of the AUCs of the observers. Ideally, the system would be evaluated on an independent test set [18]. Nonetheless, the study did demonstrate that, if a CDSS-T aid could achieve this level of performance in the clinical cases, it would have the potential of improving the treatment response assessment of bladder cancer for a wide range of physicians of different specialties, varied levels of experience, and from different institutions.

In our future work, we will enlarge the dataset and use an independent dataset for both the CDSS-T system evaluation and the observer study. We will improve the performance of the CDSS-T system by integrating radiomics with clinical data and molecular and histopathology biomarkers and by incorporating different deep learning models. We will also perform observer study on a larger scale to further test the effectiveness of the computerized decision support system.

Although the results of this observer study shows that our CDSS-T system is promising as an aid to physicians for bladder cancer treatment response assessment, extensive work is still required before translation to the clinic. First, further improvement of its performance is needed as described above. Second, the processing pipeline has to be automated, including the AL-CALS system for auto tumor segmentation [10] and radiomic feature extraction. Third, the CDSS-T system will need to undergo rigorous validation of its generalizability with a wide range of patient cases from multiple sites. Finally, well-controlled clinical trial should be conducted by incorporating it into a real-world workflow to evaluate its effectiveness and efficiency as a preparatory step before clinical translation.

## 5. Conclusions

In conclusion, our study demonstrated that the computerized decision support system (CDSS-T) has the potential to improve the diagnostic accuracy in assessing the complete response of muscle-invasive bladder cancer to neoadjuvant chemotherapy prior to radical cystectomy. The use of CDSS-T aid has resulted in improved and more consistent diagnostic performance among the physicians from multiple institutions and multiple specialties.

## Figures and Tables

**Table 1:** Characteristics of the patients and cancers used in the observer study.

Characteristics	Notes	Detail		Total Number
Patient gender and age	100 males	Mean: 63 years Range: 43–84 years		123 patients
	23 females	Mean: 63 years Range: 37–82 years		
Average maximum diameter (mm)	Completely responding cancers (T0 stage)	Pre-treatment: 30.1 Post-treatment: 14.3		157 cancer pairs
	Incompletely responding cancers (>T0 stage)	Pre-treatment: 43.0 Post-treatment: 31.2		
Cancer stage		Pre-treatment	Post-treatment	157 cancer pairs
	T0	0	40	
	T1	8	37	
	T2	76	23	
	T3	63	38	
	T4	10	19	

**Table 2:** Observers of different specialties and from different institutions.

Specialty	Observer Number	Proficiency	Institution			
			UM	UI	PSU	TH
Abdominal Radiologist	5	Experienced	4	-	-	1
Diagnostic Radiology Resident	4	Inexperienced	4	-	-	-
Urologist	1	Experienced	1	-	-	-
Oncologist	5	Experienced	2	2	1	-
Medical Student	1	Inexperienced	-	-	1	-
Neurology Fellow	1	Inexperienced	1	-	-	-

UM: University of Michigan, UI: University of Iowa, PSU: Pennsylvania State University, TH: Tokuda Hospital, Sofia.

**Table 3:** AUCs of observers with and without CDSS-T. The standard deviation of the AUCs of 17 observers was calculated for both the without CDSS-T and with CDSS-T conditions.

Observer #	AUC without CDSS-T	AUC with CDSS-T	Individual *p* Value
1	0.74	0.77	0.155
2	0.75	0.77	0.260
3	0.73	0.76	0.013 *
4	0.74	0.77	0.128
5	0.76	0.80	0.010 *
6	0.74	0.74	0.861
7	0.76	0.77	0.541
8	0.73	0.75	0.135
9	0.78	0.81	0.191
10	0.74	0.76	0.244
11	0.67	0.73	0.014 *
12	0.75	0.79	0.095
13	0.72	0.77	0.027 *
14	0.65	0.76	0.020 *
15	0.67	0.78	0.003 *
16	0.76	0.79	0.026 *
17	0.73	0.76	0.083
Mean AUC	0.73	0.77	0.002 *^,$^
Standard Deviation	0.04	0.02	-

* Statistically significant difference at *p* < 0.05 level. $ Obtained from MRMC analysis.

**Table 4:** Performance comparisons with and without CDSS-T for easy and difficult subsets.

	AUC of CDSS-T	Average AUC without CDSS-T	Average AUC with CDSS-T	*p* Value	# of Physicians
Easy Subset	0.88	0.80	0.84	0.016 *	17 physicians
Difficult Subset	0.67	0.58	0.62	0.148	
Easy Subset	0.88	0.83	0.85	0.033 *	9 radiologists
Difficult Subset	0.67	0.59	0.61	0.379	
Easy Subset	0.88	0.78	0.84	0.051	5 oncologists
Difficult Subset	0.67	0.57	0.63	0.009 *	

* Statistically significant difference at *p* < 0.05 level.

**Table 5:** Performance comparison between experienced and inexperienced observers for the total of 157 lesion pairs.

	AUC of CDSS-T	Average AUC without CDSS-T	Average AUC with CDSS-T	*p* Value	# of Physicians
Experienced Physicians	0.80	0.73	0.77	0.007 *	5 abdominal radiologists, 1 urologist, and 5 oncologists
Inexperienced Physicians		0.73	0.77	0.019 *	5 residents and 1 medical student
Experienced Radiologists		0.75	0.77	0.060	5 abdominal radiologists
Inexperienced Radiologists		0.74	0.77	0.007 *	4 radiology residents
UM Experienced Radiologists		0.75	0.77	0.018 *	4 abdominal radiologists from UM
UM Inexperienced Radiologists		0.74	0.77	0.007 *	4 radiology residents from UM

* Statistically significant difference at *p* < 0.05 level.

**Table 6:** Performance comparison between observers from different specialties for the total of 157 lesion pairs.

	AUC of CDSS-T	Average AUC without CDSS-T	Average AUC with CDSS-T	*p* Value	# of Physicians
Radiologists	0.80	0.75	0.77	0.014 *	9
Urologist		0.74	0.76	0.244	1
Oncologists		0.71	0.77	0.011 *	5
Medical Student		0.65	0.76	0.020 *	1
Neurology Fellow		0.73	0.76	0.083	1

* Statistically significant difference at *p* < 0.05 level.

**Table 7:** Performance comparison between observers from different institutions for the total of 157 lesion pairs.

	AUC of CDSS-T	Average AUC without CDSS-T	Average AUC with CDSS-T	*p* Value	# of Physicians
UM Physicians	0.8	0.74	0.77	0.002 *	12
TH Physician		0.74	0.74	0.861	1
PSU Physicians		0.69	0.76	0.117	2
UI Physicians		0.72	0.78	0.326	2
UM Oncologists		0.71	0.76	0.071	2
PSU Oncologist		0.72	0.77	0.027 *	1
UI Oncologists		0.72	0.78	0.326	2

* Statistically significant difference at *p* < 0.05 level.

**Table 8:** Diagnostic performance in terms of AUC of physicians without and with the CDSS-T aid for the assessment of complete response to neoadjuvant chemotherapy on the first 51 cases in each observer’s individually randomized reading list. The iMRMC package provided standard deviation value along with each AUC. The mean and standard deviations of AUC values for the 17 observers with and without CDSS-T for both the original and the repeated evaluations were calculated.

Observer #	AUC Original Evaluation		AUC Repeated Evaluation	
	Without CDSS-T	With CDSS-T	Without CDSS-T	With CDSS-T
1	0.75 ± 0.08	0.76 ± 0.08	0.8 ± 0.07	0.79 ± 0.07
2	0.88 ± 0.05	0.91 ± 0.04	0.88 ± 0.05	0.92 ± 0.03
3	0.65 ± 0.10	0.72 ± 0.10	0.67 ± 0.10	0.72 ± 0.09
4	0.71 ± 0.09	0.71 ± 0.09	0.69 ± 0.09	0.71 ± 0.08
5	0.70 ± 0.07	0.78 ± 0.06	0.82 ± 0.06	0.83 ± 0.05
6	0.82 ± 0.07	0.85 ± 0.07	0.81 ± 0.07	0.81 ± 0.07
7	0.75 ± 0.08	0.77 ± 0.08	0.84 ± 0.05	0.87 ± 0.05
8	0.74 ± 0.09	0.77 ± 0.08	0.81 ± 0.08	0.8 ± 0.08
9	0.81 ± 0.06	0.85 ± 0.05	0.8 ± 0.06	0.85 ± 0.05
10	0.79 ± 0.08	0.84 ± 0.07	0.8 ± 0.07	0.87 ± 0.07
11	0.65 ± 0.08	0.75 ± 0.08	0.73 ± 0.07	0.78 ± 0.07
12	0.81 ± 0.07	0.85 ± 0.07	0.75 ± 0.08	0.76 ± 0.07
13	0.81 ± 0.06	0.89 ± 0.04	0.77 ± 0.07	0.83 ± 0.06
14	0.59 ± 0.10	0.82 ± 0.07	0.69 ± 0.10	0.81 ± 0.07
15	0.73 ± 0.07	0.88 ± 0.06	0.64 ± 0.10	0.83 ± 0.07
16	0.86 ± 0.05	0.93 ± 0.03	0.87 ± 0.05	0.87 ± 0.05
17	0.63 ± 0.08	0.69 ± 0.08	0.68 ± 0.10	0.76 ± 0.09
Mean AUC	0.75	0.81	0.77	0.81
Standard Deviation	0.08	0.07	0.07	0.06
**Statistical significance in the difference of AUC:**				
AUC (orig.without) versus AUC (orig.with): *p* = 0.003 *				
AUC (repeat.without) versus AUC (repeat.with): *p* = 0.006 *				
AUC (orig.without) versus AUC (repeat.without): *p* = 0.217				
AUC (orig.with) versus AUC (repeat.with): *p* = 0.692				

* Statistically significant difference at *p* < 0.05 level.

## Data Availability

Data available upon request.

## Abbreviations

AUC: area under curve; CAD: computer-aided diagnosis; CDSS-T: computerized decision support system for bladder cancer treatment response assessment; CTU: CT urography; DL-CNN: deep-learning convolutional neural networks; IRB: Institutional Review Board; MRMC: multi-reader, multi-case; PSU: Pennsylvania State University; RECIST: Response Evaluation Criteria in Solid Tumors criteria; ROC: receiver operating characteristics; ROI: regions of interest; TH: Tokuda Hospital, Sofia; UI: University of Iowa; UM: University of Michigan.
